# A Multiplex PCR/LDR Assay for the Simultaneous Identification of Category A Infectious Pathogens: Agents of Viral Hemorrhagic Fever and Variola Virus

**DOI:** 10.1371/journal.pone.0138484

**Published:** 2015-09-18

**Authors:** Sanchita Das, Mark S. Rundell, Aashiq H. Mirza, Maneesh R. Pingle, Kristi Shigyo, Aura R. Garrison, Jason Paragas, Scott K. Smith, Victoria A. Olson, Davise H. Larone, Eric D. Spitzer, Francis Barany, Linnie M. Golightly

**Affiliations:** 1 Department of Medicine, Division of Infectious Diseases, Weill Medical College of Cornell University, New York, New York, United States of America; 2 Department of Microbiology and Immunology, Weill Medical College of Cornell University, New York, New York, United States of America; 3 United States Army Medical Research Institute of Infectious Diseases, Frederick, Maryland, United States of America; 4 Integrated Research Facility, Division of Clinical Research, NIAID, NIH, Fort Detrick, Maryland, United States of America; 5 Poxvirus Team, Poxvirus and Rabies Branch, Division of High Consequence Pathogens and Pathology, National Center of Emerging Zoonotic and Infectious Diseases, Centers for Disease Control and Prevention, Atlanta, Georgia, United States of America; 6 Department of Pathology and Laboratory Medicine, Weill Medical College of Cornell University, New York, NY, United States of America; 7 Department of Pathology, Stony Brook University Medical Center, Stony Brook, New York, United States of America; Division of Clinical Research, UNITED STATES

## Abstract

CDC designated category A infectious agents pose a major risk to national security and require special action for public health preparedness. They include viruses that cause viral hemorrhagic fever (VHF) syndrome as well as variola virus, the agent of smallpox. VHF is characterized by hemorrhage and fever with multi-organ failure leading to high morbidity and mortality. Smallpox, a prior scourge, has been eradicated for decades, making it a particularly serious threat if released nefariously in the essentially non-immune world population. Early detection of the causative agents, and the ability to distinguish them from other pathogens, is essential to contain outbreaks, implement proper control measures, and prevent morbidity and mortality. We have developed a multiplex detection assay that uses several species-specific PCR primers to generate amplicons from multiple pathogens; these are then targeted in a ligase detection reaction (LDR). The resultant fluorescently-labeled ligation products are detected on a universal array enabling simultaneous identification of the pathogens. The assay was evaluated on 32 different isolates associated with VHF (ebolavirus, marburgvirus, Crimean Congo hemorrhagic fever virus, Lassa fever virus, Rift Valley fever virus, Dengue virus, and Yellow fever virus) as well as variola virus and vaccinia virus (the agent of smallpox and its vaccine strain, respectively). The assay was able to detect all viruses tested, including 8 sequences representative of different variola virus strains from the CDC repository. It does not cross react with other emerging zoonoses such as monkeypox virus or cowpox virus, or six flaviviruses tested (St. Louis encephalitis virus, Murray Valley encephalitis virus, Powassan virus, Tick-borne encephalitis virus, West Nile virus and Japanese encephalitis virus).

## Introduction

Viral hemorrhagic fever is a febrile syndrome associated with vascular damage caused by RNA viruses of the families: *Filoviridae* [*Ebolavirus* and *Marburgvirus*], *Arenaviridae* [Lassa fever virus (LASV)], *Bunyaviridae* [Rift Valley fever virus (RVFV) and Crimean Congo hemorrhagic fever virus (CCHFV)], and *Flaviviridae* [Yellow fever virus (YFV), and dengue virus (DENV)]. Most are zoonotic, vector-borne, and may cause sporadic, unanticipated, and devastating outbreaks in endemic areas [1]. The Center for Disease Control and Prevention (CDC) has designated *Filoviridae* and *Arenaviridae* as Category A due to their ease of transmission, high mortality, risk to national security, and potential for causing public panic and social disruption. Many agents of VHF are designated as emerging or reemerging pathogens and threaten not only their traditional areas of endemicity in developing countries but new territory in other countries as well [2–5]. There are few preventative vaccines, and clinical management is largely supportive due to the paucity of effective chemotherapeutic agents. The case fatality ratio for outbreaks of the filoviruses in Africa has ranged from approximately 36–90% and 83–90% for Marburg and Ebola, respectively [6–8]. Furthermore, the infection of skilled and traditional healers tending the sick complicates care and control measures while abetting nosocomial transmission and the spread of disease [2, 9, 10]. The 2014 West African Ebola outbreak, which is the largest recorded thus far, exemplifies these difficult infection control issues [11–13]. The filoviruses, CCHFV, and LASV require the highest level of laboratory containment (containment level 4 or biosafety level 4), however, few such facilities are present in resource-poor endemic countries [2]. The worldwide threat of VHF agents to the public health, as well as to veterinary and agricultural communities, is increasingly recognized, as is the possibility of the accidental or malicious release of some of these viruses as agents of bioterror [14, 15].

Human smallpox was eradicated over 30 years ago. Since then, vaccination to poxviruses has largely stopped, leading to a worldwide population of susceptible individuals [14, 16]. Variola virus is legally retained at only two World Health Organization (WHO) Collaborating Center repositories. Reports of covert, undeclared stocks and weaponized virus have fueled fears that variola may be reintroduced as a bioterror agent, an issue of continuing national and international concern [17–19].

Recognition of index cases with the exotic and geographically restricted VHF viruses, or eradicated smallpox, depends upon the clinical suspicion and diagnostic acumen of first-line physicians [20, 21]. VHF infections due to endemic natural outbreaks, infection in returning travelers, or suspected acts of bioterrorism continue to challenge public health and clinical laboratories. Effective infection control and the implementation of public health containment plans require rapid and effective diagnostic tests. [1, 22].

Rapid detection and specific pathogen identification are key to the control of outbreaks due to VHF and variola viruses. In addition, rapid screening of a large number of samples can be anticipated as was the case in the 2001 anthrax outbreak [15]. There are several molecular detection assays that have been developed for rapid detection of VHF viruses, the majority of which are based on real-time PCR [23–26]. These assays have improved the sensitivity and the turn-around time for detection of VHF, while significantly reducing the biohazard potential of cultivating these organisms in the laboratory. However, real-time PCR assays often have limited multiplexing capabilities and require several assays to be run for detection of multiple etiologic agents [15, 27]. An additional concern is mutation in these viruses resulting in the alteration of protein sequences and targets used for molecular detection. Sequencing has established that in the recent West African Ebola outbreak, isolates from Sierra Leone had genomes that varied from PCR probes used for four separate assays used for EBOV and pan-filoviral diagnostics [19]. Mutations could potentially impact diagnosis due to mismatch between target and primer/probe, not only in current assays, but also in those that are in the process of being released [19, 28, 29].

Multiplex detection of several viruses using real-time PCR strategy is limited by the choice of fluorescent dyes and their spectral overlap [30, 31]. Palacios et al. have described a multiplex assay for detecting VHF viruses that utilizes PCR primers containing unique mass tags that are then detected by a mass spectrometer [24]. A comprehensive molecular detection panel for high-throughput identification of these viruses, using a single cycling protocol and fluorescent technology, would be a useful addition to current techniques available for molecular identification of these pathogens [27, 32, 33]. PCR/LDR is a versatile technique that has been used in the detection of pathogens in clinical as well as environmental samples [34–36]. We have previously reported a PCR/LDR universal array-based technique for the simultaneous detection and identification of all four serotypes of DENV and West Nile virus from serum and plasma samples as well as from mosquito pools [37, 38]. Here we describe use of this technology for the multiplex detection and identification of seven VHF viruses (ebolavirus [Zaire, Sudan and Reston ebolaviruses], MARV, LASV, CCHFV, RVFV, YFV, DENV) and two orthopoxviruses [variola virus (VARV) and vaccinia virus (VACV)] in a single assay.

## Methods

### Ethics Statement

This study was performed in accordance with a protocol approved by the Institutional Review Board of the Weill Medical College of Cornell University.

### Viral Isolates

Vaccine strains of RVFV (MP12) and YFV (17D) were kindly provided as a gift by Dr. Robert Tesh, University of Texas Medical Branch, Galveston, TX. Inactivated viral culture supernatants of ebolavirus [Zaire virus (EBOV), Sudan virus (SUDV), and Reston virus (RESTV)] (n = 4), MARV (n = 3), CCHFV (n = 3), RVFV (n = 1) and LASV (n = 1) were obtained from the United States Army Medical Research Institute of Infectious Diseases, Ft. Detrick, Maryland. Genomic DNA from VACV virus (n = 6) was obtained from NIH Biodefense and Emerging Infections Research Resources Repository, NIAID, NIH. The two target amplicons of VARV [amplicon 1 (RAP94): 421bp; nt: 77,877–78,298 and amplicon 2 (RPO147): 485bp; nt: 82,372–82,856] representing all VARV sequence variants for these genetic regions [39] were obtained from the CDC Poxvirus and Rabies Branch, Centers for Disease Control and Prevention, Atlanta, GA with the permission of the WHO and in accordance with all applicable regulations [40]. Details of viral cultures and nucleic acid used in the study are provided in Tables 1 and 2.

**Table 1:** Details of viral cultures and nucleic acids used in the study: RNA viruses.

Viral isolate / Strain	Source/Year	Geographic Location	Accession Number
**Ebolavirus**			
*Zaire ebolavirus*’95	Human/1995	Democratic Republic of Congo	JQ352763
*Zaire ebolavirus*’76	Human/1976	Democratic Republic of Congo	NC_002549
*Reston ebolavirus*	Primate/1989	USA	NC_004161
*Sudan ebolavirus*	Human/1976	Sudan	AF173836*
**Crimean Congo hemorrhagic fever**			
Hy-13	*Hyalomma asiaticum* tick/1968	China	AY900145
UG3010	Human/1956	Democratic Republic of Congo	AY900143
ArD8194	*H*. *truncatum/*1969	Senegal	DQ211626
**Marburg virus**			
Musoke	Human/1980	Kenya	M92834
RAVN	Human/1987	Kenya	EF490232
Ci67	Human/1967	Germany	EF446132
**Rift Valley fever virus**			
ZH501	Human/1977	Egypt	DQ380200.
MP-12	Vaccine strain	-	Z30318
**Yellow fever virus**			
17D	Vaccine strain	-	NC_002031
**Dengue virus**			
DENV-1,-2,-3,-4	Hawaii, New Guinea C, Philippines H87, Philippines H241	Standard strains	KM204119, KM204118, AJ320521, FJ439174
**Lassa virus**			
Lassa-Jossiah	Human/1976	Sierra Leone	NC_004297

* The exact sequence of the stock used is not known. The GenBank sequence of the parent strain is indicated.

**Table 2:** Details of viral cultures and nucleic acids used in the study: DNA viruses.

Viral isolate / Strain	Source/Year	Geographic Location	Accession Number
***Variola* virus PCR amplicons** †			
BSH75_banu	Human/1975	Bangladesh	DQ437581
BSH74_nur	Human/1974	Bangladesh	DQ441420
BSH74_sol	Human/1974	Bangladesh	DQ441421
BSH74_shz	Human/1974	Bangladesh	DQ441422
CHN48_horn	Human/ 1948	China	DQ437582
GER58_hdlg	Human/1958	Heidelberg, Germany	DQ437584
V73-175	Human/1973	Nepal	DQ437588
SAF65_102	Human/1965	Natal, South Africa	DQ441435
**Vaccinia virus DNA** ‡			
Lister (Elstree)	BEI Resources (ATCC)		AY678276
Modified Vaccinia Ankara	BEI Resources (ATCC)		U94848
Lederle-Chorioallantoic	BEI Resources (ATCC)		AM501482
New York City Board of Health (NYCBH) Wyeth, calf adapted	BEI Resources (ATCC)		JN654986
Western Reserve (WR) NIAID,Tissue culture adapted	BEI Resources (ATCC)		AY243312
International Health Division (IHD)	BEI Resources (ATCC)		KC201194

†PCR amplicons from RAP94 and RPO147 gene segments (421 and 485 bp respectively) were obtained from the Poxvirus Program, Centers for Disease Control and Prevention, Atlanta, GA. See text and reference [39] for further details about the VAR strains used for PCR amplification.

‡Complete information about the VACV virus DNA is available at the ATCC’s Biodefense and Emerging Infections Research Resources Repository (BEI) website at http://www.beiresources.org/Catalog/tabid/248/Default.aspx.

As previously described, culture supernatants from standard isolates of the four serotypes of DENV were employed [37]. The following viruses or nucleic acids were used as controls: DNA from cowpox and monkeypox viruses obtained from ATCC; St. Louis encephalitis virus (strain MSI-7), Murray Valley Encephalitis virus (strain OR2), Powassan virus (strain M11665), Tick-borne encephalitis virus (strain K23), West Nile virus (NAT-positive plasma samples from blood donors) and Japanese encephalitis virus (strain SA-14-14-2) [38].

### Oligonucleotide Design

Virus-specific primers were designed as described previously [37, 38]. Briefly, after alignment of the sequences, areas with relative conservation among different virus strains were chosen for each virus group and, where possible, for several viral groups (*Filoviridae*, *Flaviviridae* and *Poxviridae*) so as to achieve maximum strain coverage with the least number of primers. Primer sets were designed to simultaneously amplify two different regions in each virus or viral group: *NP* and *L* genes of ebolaviruses (EBOV, SUDV and RESTV) and MARV, *S* segment of CCHFV, *M* and *S* segments of RVFV, *L* segment of LASV, *NS5* regions of DEN and YF, and the *RNA pol* (RAP94 and RPO147) genes of VACV and VARV; a total of 57 primers. The amplicons were ~500 bp in length (range 399–685 bp). The primer sequences contained no more than three degenerate positions and had a melting temperature of around 72–75°C (S1 Table) [37, 38].

LDR primers were chosen in two to three different conserved regions within each of the two PCR amplicons for the different virus groups. The primers were designed with the intent of achieving the highest possible strain coverage in all the different viruses as well as to differentially identify them individually. A total of 250 LDR primers were designed, with melting temperatures between 75 and 80°C; degenerate bases (no more than three in each primer) were introduced, where required, to account for sequence variations. A complete list of all LDR primers is provided in S2 Table. The PCR and LDR primers were obtained from Integrated DNA Technology, Coralville, IA.

The PCR and LDR primers for each of the target regions for all the virus groups were evaluated in separate assays individually. This was performed to evaluate satisfactory signal detection from each of the regions selected. Primers that failed to produce either PCR amplicon or generated less than two LDR products were replaced, and new primers were designed. The LDR primers were designed such that they were able to differentiate between the three species of ebolavirus tested (EBOV, SUDV and RESTV). The poxvirus primers were designed such that they were able to discriminate between VARV and VACV and would not cross-react or produce false positive signals with other *Orthopoxvirus* species (cowpox and monkeypox viruses). The flavivirus primers were designed to identify and distinguish DENV and YFV without cross-reactivity with a panel of flaviruses.

### Nucleic Acid Preparation and PCR/LDR Assay

**Fig 1:**
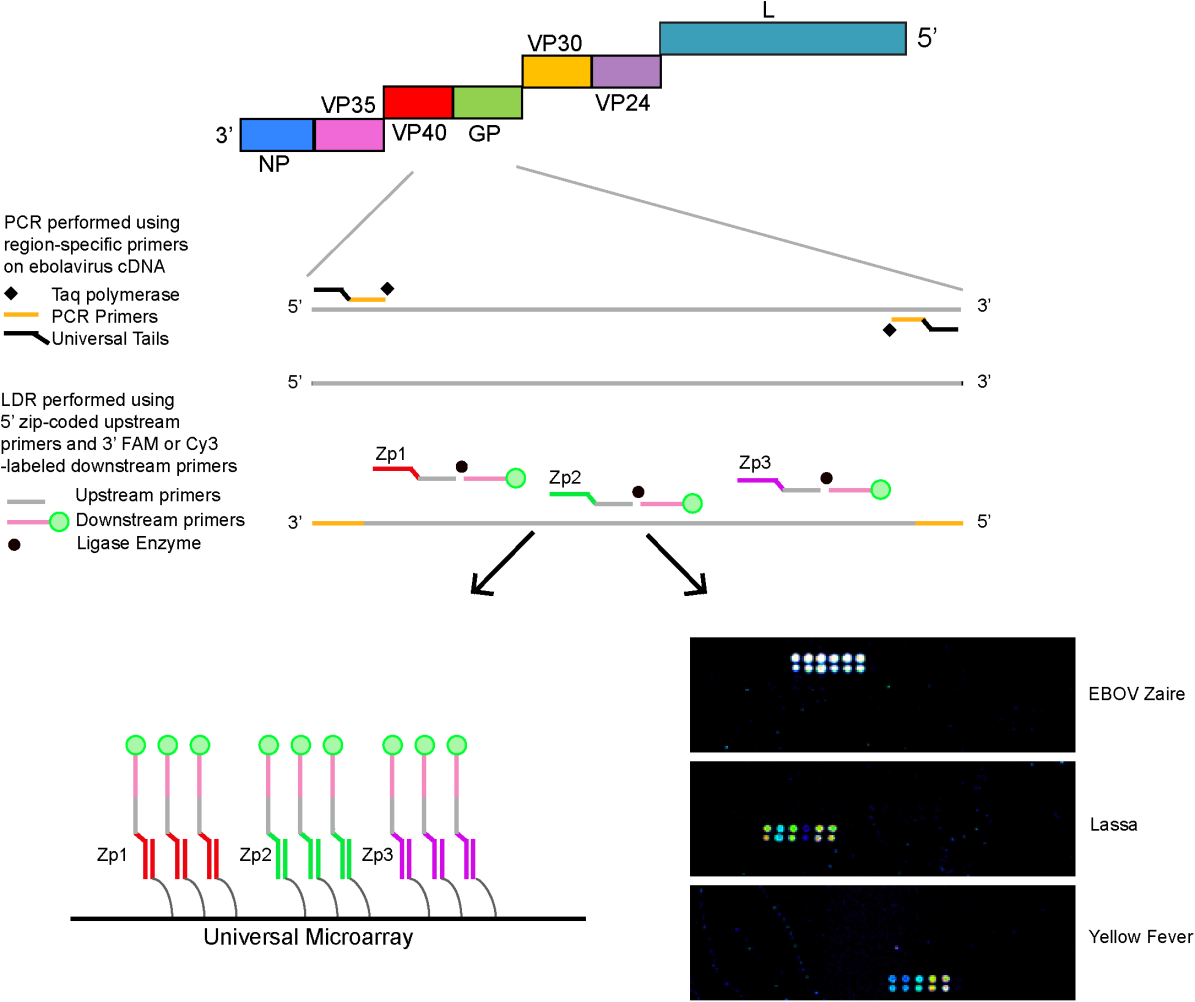
Schematic of the PCR/LDR assay for detection of VHF viruses. For each virus (ebolavirus is shown as a representative virus), 1–2 different regions are amplified by RT-PCR using forward and reverse primers, each with minimal degeneracy and all containing universal tails to prevent the formation of primer dimers. Cy-3 labeled downstream LDR primers and single base-discriminating upstream primers with unique zip-code complements (20-30-mers) are targeted to specific sequences/SNPs within the PCR amplicons. Ligation of two adjacent oligonucleotides annealed to a complementary DNA target occurs in the presence of thermostable ligase only if the nucleotides are perfectly matched at the junction [54, 55]. The zip-code complements on the 5’ end of fluorescently labeled LDR products anneal to specific complementary zip-code addresses on a universal array [56, 57]. A positive signal on the universal array is detected as a fluorescent spot. Primers for the ligation reaction were designed targeting 2 or 3 areas within each PCR amplicon. Each virus could produce a maximum of six ligation products, except for VAR and VACC, for which there were a maximum of 5 each. The detection of 2 or more ligation products was required for the detection and identification of a virus. Representative arrays that detect and identify *Ebola Zaire*, Lassa and Yellow fever viruses are shown.

Nucleic acid extraction and one-step RT-PCR amplification (OneStep RT-PCR kit; Qiagen, Valencia, CA), ligase detection reaction (LDR), and universal array were performed as described previously [37, 38], with the exception that one-step RT-PCR was performed in a 25 μl final volume using 5 μl of template RNA or DNA. Fig 1 explains the PCR/LDR assay design and detection protocol. To provide a certain degree of redundancy two regions of each virus were amplified, and primers for the subsequent ligation reaction were designed targeting 2 or 3 areas within each PCR amplicon. Consequently, up to six ligation products (only 5 ligation products in the case of VARV and VACV) can be generated for each virus to be detected. However, not all ligation products are required to be present to unambiguously detect and identify each of the viruses. The presence of any 2 ligation products for a given virus, either two ligation products from a single PCR amplicon or at least one ligation product from each of the two amplicons, was considered sufficient for identification. Ligation products bear zip-code sequences and were detected by hybridization to a universal array bearing complementary zip-codes. A signal was considered positive for ligation if the intensity of the corresponding zip-code spot was at least 10-fold higher than the overall average background intensity of the array as determined by the ScanArray Express v 4.0 (Perkin Elmer, MA). No-template controls provided no PCR amplicons and consequently no positive signals for any ligation products.

The analyses were repeated in at least 2 different experiments with the exception of *Sudan ebolavirus* for which there is a single experiment. We included the following geographic and genotypic variants: four different strains of three species of ebolavirus (SUDV, RESTV, EBOV); standard strains of DENV serotypes 1–4; VARV (8 genotypically variant isolates of *V*. *major* virus); and CCHFV (3 isolates; Hy-13 from China, and UG3010 and ArD8194 from the Democratic Republic of the Congo and Senegal, respectively).

### Preparation of RNA Standards for Determination of LOD

Synthetic RNA fragments of the viruses were prepared for LASV, RVFV, and YFV, MARV and CCHFV [24]. The target regions of LASV, RVFV and YFV viruses to be detected were amplified by RT-PCR, purified using QIAquick PCR purification kit (Qiagen, Inc., Valencia, CA), and cloned into the expression vector pGEM T Easy (Promega, Madison, WI) containing the T7 promoter region. The plasmids were purified, and the presence of complete inserts was confirmed by sequencing the inserts using vector-specific primers. Target regions for MAR and CCHF were synthetically constructed and inserted into the pIDT Blue vector (Integrated DNA Technologies Coralville, IA). The complete inserts of the target regions thus generated were linearized and *in vitro* transcribed using the mMessage *in vitro* transcription kit (Ambion, Austin, TX). Following DNAse treatment, the synthetic RNA was purified using an RNeasy column (Qiagen, Inc., Valencia, CA). The quantity of RNA generated for each transcript was determined using the Ribogreen® RNA Quantitation Kit (Molecular Probes, Eugene, OR) following the manufacturer’s instructions.

### Limit of Detection

The limit of detection (LOD) of the different viruses was determined in the following manner. First, 10-fold serial dilutions of the synthetically prepared, previously quantified, RNA transcripts of LASV, RVFV, YFV, MARV and CCHFV (1x10^10^ to 1x10^0^ copies/ml) were used in the PCR reaction to determine the limit of detection. Second, the quantified VACV virus DNA stocks obtained from BEI Resources were serially diluted in nuclease-free water and 5 μl of each of the DNA dilutions was used in the PCR reaction to determine limit of detection. Third, serial dilutions (ten-fold) of viral culture stocks were prepared for EBOV (*Zaire ebolavirus* ‘95) from inactivated standard stock cultures. Dilutions were prepared in Dulbecco’s minimum essential medium (DMEM) supplemented with 10% fetal bovine serum (Invitrogen, Carlsbad, California). RNA was extracted from 140 μl of each dilution, and RT-PCR-LDR/universal array was used to determine the analytical sensitivity of the assay.

## Results

### Evaluation of the Assay Using Viral Strains

**Fig 2:**
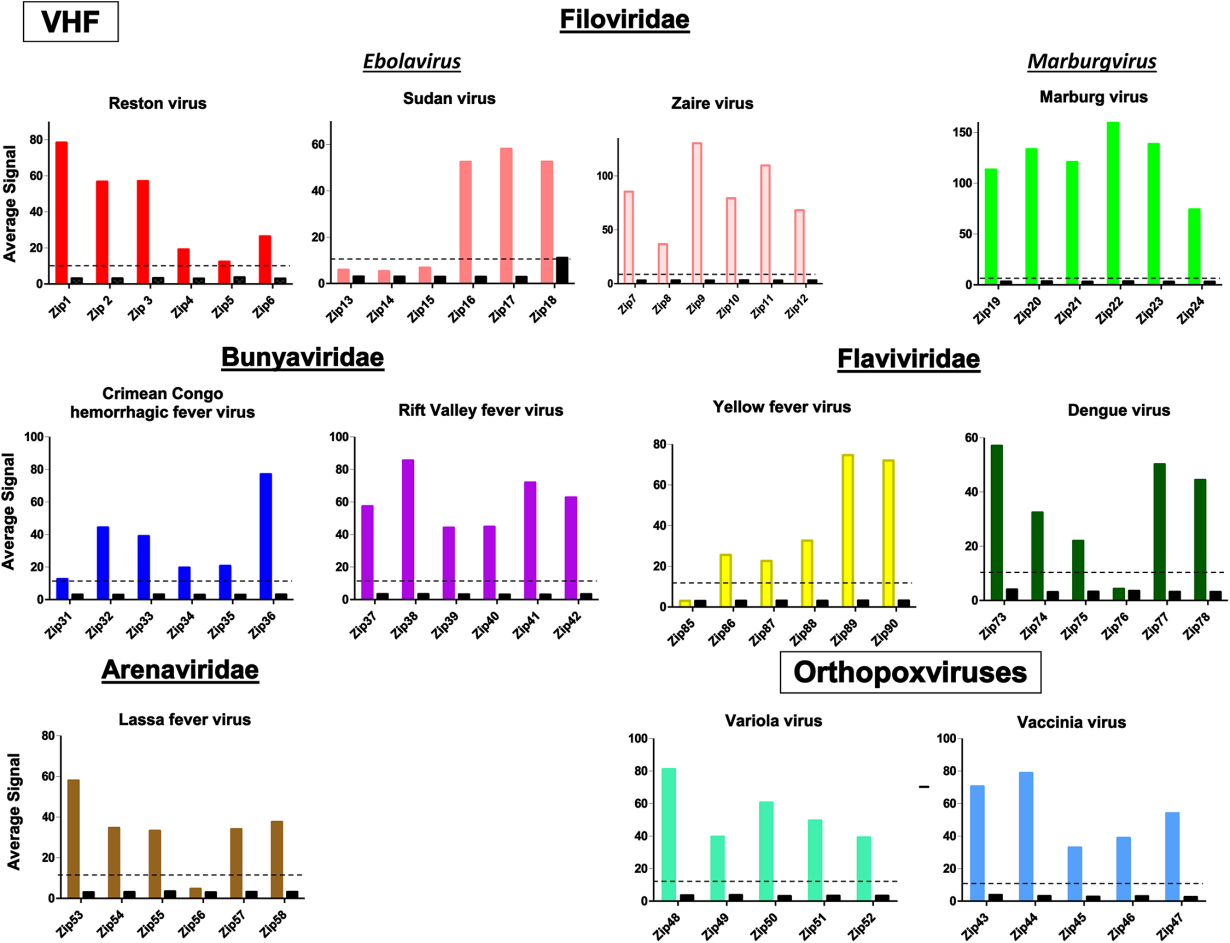
Comparison of universal array profile of viral RNA/DNA tested for the corresponding zip-codes. Normalized average signal intensity for the zip-codes assigned to each virus are presented. The color bars are the signals obtained with the indicated virus (positives). The black bars are the signals produced by the other ten viruses. A signal was considered positive if the intensity of the zip-code spot was at least 10-fold higher than the uniform background level of fluorescence of the array slide. Although a few other viruses produced low-level positive signals for zip18, this did not result in any false positive results since positive signals from at least two addresses was required for a positive identification. In the future, this issue would be rectified by switching to a different zip-code. The average signal intensity for the positives ranged from 31.2 to 123.4, depending on the virus. The average signal intensity for the negatives ranged from 0.3 to 6.2, thus they were not considered positive signals.

The PCR/LDR/universal array was validated on 53 different samples including viral culture filtrates, vaccine strains of viruses, nucleic acid from VACV viruses, and PCR amplicons <500bp in size of VARV. Fig 2 depicts the ability of the primers to detect and differentiate the viruses from each other. The PCR-LDR assay was able to detect all 32 strains, representing 9 different viruses that were included in the assay. No signals were observed for blank no-template controls and no false positives were seen with 8 other related viruses (data not shown). The assay was designed to provide redundancy and account for sequence drift in the viruses to be examined. Two regions were targeted for PCR amplification and between 2–3 regions within each PCR amplicon were targeted for the ligation reaction such that there were a maximum of 5 or 6 possible signals for each DNA or RNA virus respectively, but only 2 positive signals were required to confirm the identification of any given virus. Using this criterion, the assay detected all 11 viruses/strains with no false identification. Seven of the viruses generated positive signals for all LDR targets (5/5 or 6/6) and three were positive at 5/6 targets (see Fig 2). We observed only 3 positive signals in *Sudan ebolavirus* because only one PCR region was successfully amplified. None of these signal drop-outs affected identification of the viral species and were expected given the inherent variability of RNA viruses.

Due to restrictions on the possession of VARV and its nucleic acid, DNA extraction and PCR amplification of the RAP94 and RPO147 genes was performed at the CDC. All 5 possible LDR signals from the 8 strains representative of sequence variants in the CDC repository were positive. The experiments were performed in accordance with the regulations and permission of the WHO. All 5 possible signals were detected for the VACV strains tested. No false-positive reactions were observed. The primers for VACV did not produce false signals with VARV DNA and vice versa, nor did either set of primers interact with cowpox virus or monkeypox virus DNA (data not shown).

### Limit of Detection

The limits of detection using either synthetically transcribed RNA or dilutions of whole virus are shown in Table 3. The LODs for CCHFV and MARV viruses were 190 and 53 RNA copies/ml respectively. The LODs for RVFV and LASV were 7.6 and 100 RNA copies/ml, respectively. The LODs for the whole virus dilutions tested for EBOV and DENV were 1000 FFU/ml and 1PFU/ml respectively. The calculated limits of detection per PCR reaction for EBOV and DENV viruses were <10 copies/PCR reaction. The LOD for the DNA virus, VACV was found to be 10^4^ genome equivalents/reaction.

**Table 3:** The limit of detection of the PCR/LDR/Universal Array assay using *in vitro* transcribed RNA or whole virus.

Organism	RNA detected (copies/ml) ^a^	Whole Virus detected ^b^
MARV	1.9 x 10^2^	ND
CCHFV	5.3x10^1^	ND
LASV	1x10^2^	ND
RVFV	7.6x10^0^	ND
DENV	ND	1 pfu/ml
EBOV	ND	5x10^3^ FFU/ml
YFV	5.5x10^0^	ND

ND = Not determined

Pfu/ml = plaque forming units/ml

ffu/ml = focus forming units/ml.

^a^ PCR/LDR was performed on cloned RNA fragments for all viruses except EBOV and DENV while

^b^dilutions of culture supernatants were used for the latter two viruses. *Zaire ebolavirus*’95 was used for determination of LOD.

## Discussion

We describe a multiplex detection assay that can simultaneously detect and differentiate 7 VHF viruses, VARV and VACV, in a single assay and is amenable to adaptation to a high-throughput format. The PCR/LDR/universal array was validated on 53 different samples including viral culture filtrates, vaccine strain of viruses, serum specimens from patients with DENV infection, nucleic acid from VACV viruses and PCR amplicons <500bp in size of VARV. Since the viruses that cause the VHF syndrome include variant viral species with diverse geographical distributions, we attempted to include viruses from several geographic areas and viral species when applicable. The assay has some built-in redundancy, by including 6 possible signals for each virus (only 2 of which are required for identification), to account for the genetic diversity often encountered in RNA viruses. We encountered signal drop-out in some of the viral species but the assay was able to detect all viruses included in the panel. Additionally, the number of signals detected, or the fluorescent intensity of the signals generated, was robust for all samples tested.

Early stages of infection for most of these viruses are relatively non-specific. For some viruses, such as RFV and Lassa, the classical hemorrhagic manifestations may be absent, making accurate and timely diagnosis challenging. A multiplexed detection assay such as the PCR/LDR has a potential benefit in being able to screen for several pathogens in an endemic area or in symptomatic returning travelers. In fact, there are several multiplex assays that have been developed in the recent years to address this issue [41]. The analytical sensitivity of our assay was comparable to recently described real-time PCR based assays [41]. The PCR/LDR has the significant advantage of testing for 9 agents simultaneously and is amenable to automation. Additional targets can be added to the repertoire without compromising the performance of the assay.

The multiplex capability of PCR-LDR allowed an assay design that can distinguish viral strains that differ in geographic distribution and virulence. There are currently five recognized species of ebolavirus, two of which, *Sudan ebolavirus* and *Zaire* ebolavirus, have been consistently recognized as causing large outbreaks since 1976 with the most recent in 2014 [11, 42]. The remaining three species, *Tai Forest ebolavirus* (formerly *Cote d’Ivoire*), *Reston* and *Bundibugyo ebolaviruses* have occurred less frequently [11]. *Tai Forest ebolavirus* has been implicated in a single human infection acquired during the autopsy of a wild chimpanzee [43]. *Reston ebolavirus* causes VHF and death in primates and illness in pigs [2]. Antibody seroconversion in human contacts of infected animals has been documented but without any associated disease. Although *Reston ebolavirus* is thought to be non-pathogenic in humans, the WHO has cautioned that the effect of the infection in the immunosuppressed, pregnant women, and children is unknown [44]. Its detection therefore remains potentially clinically important. In contrast, the relatively new *Bundibugyo ebolavirus* is known to be pathogenic. The outbreak in Uganda (2007–8) resulted in 100 cases with a fatality rate of ~40% [7, 10, 42]. The PCR/LDR assay was successful in the identification of the two major outbreak associated isolates of *Zaire ebolavirus* as well as the isolates of *Sudan* and *Reston ebolaviruses* tested (Table 1). Signal dropout was noted in *Sudan ebolavirus* for all three LDR products for the nucleoprotein gene. Since 2 out of 6 signals were required for positive identification, this did not affect the sensitivity of the assay.

The assay was able to detect MARV from the initial 1967 Ugandan derived German isolate as well as the two Kenyan isolates derived from infections associated with the Kitum Cave in Kenya’s Mount Elgon National Park in 1980 and 1987 [2, 45]. Of the CCHFV S segment-defined groups (A, B and C) the assay was able to detect group A viruses from both the African and Asian clades as well as group B virus. Group C was not tested but contains a single virus isolated from a tick in Greece [46].

The assay detects all VARV genetic sequence variants in the CDC repository for the targeted regions. Comparative genomics of 45 geographically diverse VARV isolates obtained over 30 years during the smallpox era indicate low sequence diversity [39]. Hence, we anticipate that our assay would be capable of detecting the virus in the event of a bioterror attack. Since vaccination would be instituted in the event of the intentional release of variola, and vaccine strains can be spread secondarily, the assay is also designed to detect and identify VACV vaccine strains. The current version of the assay does not include specific primers for the detection of zoonotic *Orthopoxviruses*: monkeypox in particular [47], or the more clinically benign cowpox [47–49]. It was designed, however, to permit their exclusion. The modular nature of the assay allows for the expansion of the number of organisms and targets that can be identified. Future versions of the assay could be designed to include these viruses, and other hemorrhagic fever viruses such as those of South America, without compromising the sensitivity and specificity of the assay.

When our assay was initially developed, the sequence information for *Bundibugyo*, *Tai Forest*, and the 2014 *Zaire ebolavirus* species were unavailable in the public databases, and therefore their detection was not incorporated. Analysis of the *Zaire ebolavirus* implicated in the 2014 West African outbreak indicate that it would be detected and correctly identified by the assay (S1 and S2 Figs). In addition, analysis of the assay PCR primers for both the NP and L gene amplicons suggest that PCR products would be generated for both *Bundibugyo* and *Tai Forest ebolaviruses* (S1 Fig). These analyses however would need to be verified experimentally. Given the modular nature of the assay it would be relatively simple to add additional LDR primers for detection of both *Bundibugyo* and *Tai Forest ebolaviruses* to our existing assay. For example, our prototype PCR/LDR DENV assay was modified to permit the detection of an unusual strain of DENV [37].

The assay described in this study has not yet been used to detect and identify viruses directly from clinical materials nor was it directly compared to other available assays. However, we have used similar PCR/LDR assays to test for DENV in 350 acute phase serum specimens and for WNV in 142 plasma samples from blood donors. The sensitivity and specificity of the WNV assay was 100% and that of the DENV assay was 98.7% and 98.4% respectively [37, 38]. The analytical sensitivity of the assay in its present form (7–200 copies of RNA/ml of sample tested) is equivalent to <100 viral particles per assay. This is comparable to other techniques used for the detection of RNA viruses [15, 24, 41, 50]. In theory, therefore, it should detect an infection in clinical materials due to viruses in the current assay panel.

Nucleic acid extraction for this assay utilized manual extraction methods and thus required 5–6 hours. However, there are several platforms that offer high-throughput automated extraction and are in use in clinical laboratories [51, 52]. The PCR, LDR and hybridization steps have been adapted to automation in a 96-well format on a liquid handling robot in our laboratory. We have also used a 96-well bead-based array platform (BeadXpress, Illumina Inc. San Diego, CA) for downstream detection of ligation products (unpublished data, [53]). The combination of automated extraction and hybridization steps will decrease both hands-on and turn-around time for this assay which would be critical in a high volume situation such as occurred during the US anthrax attacks of 2001 [15]. Although the current version of the assay was not designed for point-of-care diagnoses, adaptations currently under investigation are designed to permit this.

PCR/LDR has a major advantage over traditional PCR based assays due to the high fidelity of the thermostable ligase enzyme; it is highly specific for a given nucleotide sequence at the site of ligation [54, 55]. The addition of a universal array, spotted with zip-code addresses, has a unique potential to recognize pathogen-specific zip-code complements appended to the LDR primers [56, 57]. This provides an additional technical advantage over existing PCR-based assays, in that it obviates the use of actual genetic sequence on the array for pathogen detection. This is especially useful for multiplexing, as a large number of genomic targets can be detected simultaneously using this array. Such multiplexing capability presents the possibility that an assay panel could be designed to target several pathogens with similar clinical features in a given endemic area, i.e., a “customized” chip. Additionally, the same array can be used for the simultaneous detection of different organisms, as positive hybridization is dependent on the chemistry of the synthesized zip-code oligonucleotides spotted on the array and their complements appended to the primers. In previous studies, we have reported the identification of 20 different bacterial species in blood cultures with a high degree of sensitivity and specificity [58].

The modular nature of this assay allows for the expansion of the number of organisms and targets that can be identified. This is especially important for identification of potentially variable genomes of viral infectious agents that may necessitate additions to the detection primers used. Additions may also be made for the detection of new hemorrhagic fever viruses as they emerge. The assay therefore adds a potential new tool to our armamentarium for the rapid high throughput detection of emerging viral pathogens and potential bioterror agents.

## Supporting Information

S1 Fig PCR amplification of 2014 *Zaire ebolavirus*. Amplicon 1 and 2 PCR primer alignments of the NP and L genes of 2014 *Zaire ebolavirus* isolates from Guinea (KJ660348) and Sierra Leone (KM233116), and isolates of *Bundibugyo ebolavirus (*NC_014373), and *Tai Forest ebolavirus* (FJ217162). *Zaire ebolavirus* 1976 (KC242801) is included to show the original alignment used for assay design. Nucleotides in red font indicate mismatches. Only relevant areas of the DNA sequence with primer binding sites are shown. Numbers indicate nucleotide positions in GenBank.
(PDF)

S2 Fig LDR identification of 2014 *Zaire ebolavirus*. Amplicon 1 and 2 LDR ligation primer alignments and ligation sites of the NP and L genes of 2014 *Zaire ebolavirus* isolates from Guinea (KJ660348) and Sierra Leone (KM233116). *Zaire ebolavirus* 1976 (KC242801) is included to show the original alignment used for assay design. Nucleotides in red font indicate mismatches. Yellow indicates the single base variant at the junction ligation site. The terminal nucleotide “T” at the 3’end highlighted in green indicates an addition to prevent quenching of fluorescence of Cy-3 dye by the adjacent guanine bases. Only relevant areas of the DNA sequence with primer binding sites are shown. Numbers indicate nucleotide positions in GenBank.
(PDF)

S1 Table PCR primers used for detection of VHF and *Orthopoxviruses*. Multiple primers were designed for each amplicon for the different viruses and are numbered; multiple primers for the same virus were required to accommodate sequence variations and these are then differentiated by the letters a, b, c and so forth. The underlined nucleotides indicate the sequence of the universal tails.
(PDF)

S2 Table LDR primers used for detection of VHF and *Orthopoxviruses*. Primer names indicate the virus detected as well as the amplicon on which the LDR primer pairs were designed. For example, the first primer was designed to detect RESTV and was designed on a nucleotide position on amplicon 1. When more than one primer pair was designed for a particular virus, they were indicated with different numbers 1,2,3 etc. When multiple primers were designed at any LDR position an alphabetical suffix is included (for example, “A” or “B”). An amino blocking group (Blk) was used at the 5’end of the upstream primers and downstream primers were labeled with Cy-3 at the 3’ end. The first 20 nucleotides of the upstream primer (underlined) are the zip-code complements.
(PDF)
