# Supplementary material for: A Multiplex PCR/LDR Assay for the Simultaneous Identification of Category A Infectious Pathogens: Agents of Viral Hemorrhagic Fever and Variola Virus
Source: PLoS One. 2015 Sep 18;10(9):e0138484. doi: 10.1371/journal.pone.0138484 (PMC4575071; doi:10.1371/journal.pone.0138484)
Supplement: S1 Fig — Amplicon 1 and 2 PCR primer alignments of the NP and L genes of 2014 Zaire ebolavirus isolates from Guinea (KJ660348) and Sierra Leone (KM233116), and isolates of Bundibugyo ebolavirus (NC_014373), and Tai Forest ebolavirus (FJ217162). Zaire ebolavirus 1976 (KC242801) is included to show the original alignment used for assay design. Nucleotides in red font indicate mismatches. Only relevant areas of the DNA sequence with primer binding sites are shown. Numbers indicate nucleotide positions in GenBank. (PDF) [file pone.0138484.s001.pdf]

Amplicon 1

```
Forward primer                 5’ CCKGAAGAGGAGACWACTGAAGCWAATGC 3’
KJ660348       ---------------881 CCGGAAGAGGAGACGACTGAAGCTAATGC 910--------------
KM233116       ---------------851 CCGGAAGAGGAGACGACTGAAGCTAATGC 880--------------
NC_014373      ---------------869 CCCGAGGAGGAAACAACAGAAGCAAATGC 898--------------
FJ217162       ---------------875 CCTGAAGAGGAGACAACAGAAGCAAATGC 904--------------
KC242801       ---------------881 CCGGAAGAGGAGACAACTGAAGCTAATGC 910--------------

Reverse Primers                  3’GYACCCTYGCAGGAGTWAATGTTGG 5’
                                3’GCACGCTGGCTGGTGTTAATGTAGG 5’
KJ660348       ---------------1455 GCACCCTCGCAGGAGTAAATGTTGG 1480----------------
KM233116       ---------------1425 GCACCCTCGCAGGAGTAAATGTTGG 1450----------------
NC_014373      ---------------1443 GCACTCTGGCTGGAGTCAATGTAGG 1468----------------
FJ217162       ---------------1450 GTACCCTGGCAGGAGTAAATGTGGG 1476----------------
KC242801       ---------------1455 GTACCCTCGCAGGAGTAAATGTTGG 1480----------------
```

Amplicon 2

```
Forward primer                 5’ ATCMTGGCACCAYACMAGTGATGATT 3’
KJ660346       -------------13406 ATCATGGCACCACACAAGTGATGATT 13432----------------
KM233116       -------------13377 ATCATGGCACCACACAAGTGATGATT 13403----------------
NC_014373      -------------13393 GTCGTGGCACCACACAAGTGACGATT 13419----------------
FJ217162       -------------13392 GTCTTGGCATCACACAAGTGATGATT 13418----------------
KC242801       -------------13406 ATCATGGCACCACACAAGTGATGATT 13432----------------


Reverse primer                 3’ GTTACGMTCAGCKGTGATGGGWGAC 5’
KJ660346      --------------13782 GTTGCGCTCAGCTGTGATGGGTGAC 13768----------------
KM233116      --------------13743 GTTGCGCTCAGCTGTGATGGGTGAC 13768----------------
NC_014373     --------------13768 ATTGAGATCTGCGGTAATGGGTGAT 13793----------------
FJ217162      --------------13769 ACTGCGATCTGCGGTAATGGGTGAC 13794----------------
KC242801      --------------13782 GTTACGCTCAGCTGTGATGGGTGAC 13807----------------
```
