# Supplementary material for: A Multiplex PCR/LDR Assay for the Simultaneous Identification of Category A Infectious Pathogens: Agents of Viral Hemorrhagic Fever and Variola Virus
Source: PLoS One. 2015 Sep 18;10(9):e0138484. doi: 10.1371/journal.pone.0138484 (PMC4575071; doi:10.1371/journal.pone.0138484)
Supplement: S2 Fig — Amplicon 1 and 2 LDR ligation primer alignments and ligation sites of the NP and L genes of 2014 Zaire ebolavirus isolates from Guinea (KJ660348) and Sierra Leone (KM233116). Zaire ebolavirus 1976 (KC242801) is included to show the original alignment used for assay design. Nucleotides in red font indicate mismatches. Yellow indicates the single base variant at the junction ligation site. The terminal nucleotide “T” at the 3’end highlighted in green indicates an addition to prevent quenching of fluorescence of Cy-3 dye by the adjacent guanine bases. Only relevant areas of the DNA sequence with primer binding sites are shown. Numbers indicate nucleotide positions in GenBank. (PDF) [file pone.0138484.s002.pdf]

Amplicon 1

```
   Allele specific upstream primer
                    5'TTGAGAAGGTTCAAAGGCAAATTCAAGTA               downstream primer
                                                  CATGCAGAGCAAGGACTGATACAATATCCAACA 3'
KJ660348    -----976 TTGAGAAGGTTCAAAGGCAAATTCAAGTACATGCAGAGCAAGGACTGATACAATATCCAACA 1037-----
KM233116    -----946 TTGAGAAGGTTCAAAGGCAAATTCAAGTACATGCAGAGCAAGGACTGATACAATATCCAACA 1008-----
KC242801    -----976 TTGAGAAGGTTCAAAGGCAAATTCAAGTACATGCAGAGCAAGGACTGATACAATATCCAACA 1037-----

   Allele specific upstream primer
                              5'CAAGGGATGCACATGGTTGCC           downstream primer
                                                   GGGCATGATGCCAACGAYGCTGT 3'
KJ660348            -----1118 CAAGGGATGCACATGGTTGCCGGACATGATGCCAACGATGCTGT 1162-----
KM233116            -----1088 CAAGGGATGCACATGGTTGCCGGACATGATGCCAACGATGCTGT 1132-----
KC242801            -----1118 CAAGGGATGCACATGGTTGCCGGGCATGATGCCAACGATGCTGT 1162-----

    Allele specific upstream primer
                         GAGGTGAACTCCTTTAAGGCTGCACTC              downstream primer
                                                   AGCTCCCTGGCCAAGCATGGAGAGTAT
KJ660348      -----1295 GAGGTGAACTCCTTCAAGGCTGCACTCAGCTCCCTGGCCAAGCATGGAGAGTAT 1348-----
KM233116      -----1265 GAGGTGAACTCCTTCAAGGCTGCACTCAGCTCCCTGGCCAAGCATGGAGAGTAT 1318-----
KC242801      -----1295 GAGGTGAACTCCTTTAAGGCTGCACTCAGCTCCCTGGCCAAGCATGGAGAGTAT 1348-----
```

Amplicon 2

```
   Allele specific upstream primer
                 5'TTGCATTTAGATATGAGTTTACAGCACCTTTTATA                  downstream primer
                                                      GAATATTGCAACCGTTGCTATGGTGTTAAGAATGT 3'
KJ660346  ---13492 TTGCATTTAGGTATGAGTTTACAGCACCTTTTATAGAATATTGCAACCGTTGCTATGGTGTTAAGAATGT 13562---
KM233116  ---13462 TTGCATTTAGGTATGAGTTTACAGCACCTTTTATAGAATATTGCAACCGTTGCTATGGTGTTAAGAATGT 13532---
KC242801  ---13492 TTGCATTTAGATATGAGTTTACAGCACCTTTTATAGAATATTGCAACCGTTGCTATGGTGTTAAGAATGT 13562---

  Allele specific upstream primer
                  5'CATTATACAATCCCACAGTGTTATATGCATGTC                  downstream primer
                                                     AGTGATTATTATAATCCACCACATAACCTCACACTGGT 3'
KJ660346   --- 13575 CATTATACAATCCCACAGTGTTATATGCATGTCAGTGATTATTATAATCCACCGCATAACCTCACACTGG 13645---
KM233116   ----13545 CATTATACAATCCCACAGTGTTATATGCATGTCAGTGATTATTATAATCCACCGCATAACCTCACACTGG 13615---
KC242801  ---- 13576 CATTATACAATCCCACAGTGTTATATGCATGTCAGTGATTATTATAATCCACCACATAACCTCACACTGG 13645---

  Allele specific upstream primer
                   5'  GGGCCTAGTTCATACAGGGGTCATATG                  downstream primer
                                                 GGAGGGATTGAAGGACTGCAACAAAAACTCT 3'
KJ660346    ---- 13670 GGGCCTAGTTCATACAGGGGTCATATGGGAGGGATTGAAGGACTGCAACAAAAACTCT 13728---
KM233116    ---- 13639 GGGCCTAGTTCATACAGGGGTCATATGGGAGGGATTGAAGGACTGCAACAAAAACTCT 13697----
KC242801    -----13670 GGGCCTAGTTCATACAGGGGTCATATGGGAGGGATTGAAGGACTGCAACAAAAACTCT 13728----
```
