# Supplementary material for: A Multiplex PCR/LDR Assay for the Simultaneous Identification of Category A Infectious Pathogens: Agents of Viral Hemorrhagic Fever and Variola Virus
Source: PLoS One. 2015 Sep 18;10(9):e0138484. doi: 10.1371/journal.pone.0138484 (PMC4575071; doi:10.1371/journal.pone.0138484)
Supplement: S1 Table — Multiple primers were designed for each amplicon for the different viruses and are numbered; multiple primers for the same virus were required to accommodate sequence variations and these are then differentiated by the letters a, b, c and so forth. The underlined nucleotides indicate the sequence of the universal tails. (PDF) [file pone.0138484.s003.pdf]

Organism	PCR primer	Sequence (5´to 3´)	Gene	Amplicon Size
FILO AMP1	ESRZ-A1-PCR-F9	GCC AAC TAC CGC AAC ACC CKG AAG AGG AGA CWA CTG AAG CWA ATG C	NP	488-599
	ERZ-A1-PCR- R9a	CCA ACT ACC GCA ACC ACC AAC ATT WAC TCC TGC RAG GGT RC		
	ES-A1-PCR-R9b	CCA ACT ACC GCA ACC ACC TAC ATT AAC ACC AGC CAG CGT GC		
	M-A1-PCR-F11	GCC AAC TAC CGC AAC ACA TAC ATG CAA TAC TTG AAC CCY CCT C		
	M-A1-PCR-R11	CCA ACT ACC GCA ACC AGC ATR GTG TTC CCA ATT AAT TTG TYC		
FILO AMP2	ESRZ-A2-PCR-F10	GCC AAC TAC CGC AAC ACA TCM TGG CAC CAY ACM AGT GAT GAT T	L	399-531
	ERZ-A2-PCR-R10a	CCA ACT ACC GCA ACC A AGT CWC CCA TCA CMG CTG AKC GTA AC		
	ES-A2-PCR-R10b	CCA ACT ACC GCA ACC AGA GGA ATA TCC CAC AGG CAC TTG TGA C		
	M-A2-PCR-F12	GCC AAC TAC CGC AAC ACC ATG GCA GAA TTA TCA ACG CGT TAC		
	M-A2-PCR-R12	CCA ACT ACC GCA ACC ACT KGG TTT TCC GTG GTC ATT TAC AAC		
RVF AMP1	RVFM-A1-PCR-F	GCC AAC TAC CGC AAC ACT TTT GTA GGC AGA TGA CAG GTG C	M	599
	RVFM-A1-PCR-R	CCA ACT ACC GCA ACC ACA YGT TGT GCA AGG CTC AAC TC		
RVF AMP2	RVFS-A2-PCR-F	GCC AAC TAC CGC AAC ACG GGC CCT GTT GTG TCT TTC TC	S	439
	RVFS-A2-PCR- R	CCA ACT ACC GCA ACC ACC TGG YRA CAG GCA CAG GTC		
CCHF AMP1	CCHF-A1-PCR-F13	GCC AAC TAC CGC AAC ACG GAC TTG TGG ACA CYT TCA CAA AC	S	446
	CCHF-A1-PCR-F15	GCC AAC TAC CGC AAC ACC TTG TGG ACA CYT TYA CAA AYT C		
	CCHF-A1-PCR-R13	CCA ACT ACC GCA ACC AGC AAG GCC TGT WGC RAC AAG TGC TAT		
	CCHF-A1-PCR-R15	CCA ACT ACC GCA ACC AAT CAT RTC TGA HAR CAT CTC TTT GAC		
CCHF AMP2	CCHF-A2-PCR-F14	GCC AAC TAC CGC AAC ACG YAG AAT CAG TGA RAT GGG WGT CTG C	S	532
	CCHF-A2-PCR-F16	GCC AAC TAC CGC AAC ACG WGC ACA RGG TGC ACA GAT TGA YAC		
	CCHF-A2-PCR-R14	CCA ACT ACC GCA ACC ATC TCA AAG ATA YCG TTG CCG CAC		
	CCHF-A2-PCR-R16	CCA ACT ACC GCA ACC ATT VCC YTT GAC GTT GTA RGC ATT		
Lassa AMP1	LassaL-A1-PCR-F1a	GCC AAC TAC CGC AAC ACC CTA AWG TCC TKC TTG TTG CAA C	L	628
	LassaL-A1-PCR-F1b	GCC AAC TAC CGC AAC ACC CCA AYG TYC TTG TWG TCG CAA C		
	LassaL-A1-PCR-R1a	CCA ACT ACC GCA ACC AGT TCC TGG GTG TTG GGT GAA C		
	LassaL-A1-PCR-R1b	CCA ACT ACC GCA ACC AGT GCA TGG GTC TTR GGR GAA C		
Lassa AMP2	LassaL-A2-PCR-F2a	GCC AAC TAC CGC AAC ACC GRA CAC ATT TRC TAC CCA RTG TTC TRC	L	487
	LassaL-A2-PCR-F2b	GCC AAC TAC CGC AAC ACC GRA CAC ATT TRC TAC CTA RTG TCT TGC		
	LassaL-A2-PCR-R2a	CCA ACT ACC GCA ACC ATG AYA CWA TCA TTG ATC AGT CAG TGG C		
	LassaL-A2-PCR-R2b	CCA ACT ACC GCA ACC ATG AYA CAA TCA TTG ATC AGT CRG TTG C		
VAR/VAC	VV-A1-PCR-F17	GCC AAC TAC CGC AAC ACG AAT TGA ATC TTA GTA AGT TTA TGA TGG TTC	RNA POL	421
	VV-A1-PCR-R17	CCA ACT ACC GCA ACC ACT TTA TTG TCT CCR AAC GTA GCT CTC		
VAR/VAC	VV-A2-PCR-F18	GCC AAC TAC CGC AAC ACC CGA TGT GTC TAC ATA AAT ATG CAA AGT C	RNA POL	485
	VV-A2-PCR-R18	CCA ACT ACC GCA ACC AAA TAT TTT CGT TAT CAA CCT TTA TAA CGT ATC		

FLAVI AMP1	DENYF-A1-PCR-F2a	GCC AAC TAC CGC AAC ACC MAG AAT GGC AAT GAC TGA CAC MAC	NS5	416
	DENYF-A1-PCR-F2b	GCC AAC TAC CGC AAC ACC ACA GAT GGC AAT GAC AGA YAC RAC Y		
	DENYF-A1-PCR-F2c	GCC AAC TAC CGC AAC ACC ACA AAT AGC YAT GAC TGA YAC YAC ACC CT		
	DENYF-A1-PCR-F2d	GCC AAC TAC CGC AAC ACC CCA GTT RGC YAT GAC AGA CAC AAC		
	DENYF-A1-PCR-F2e	GCC AAC TAC CGC AAC ACC YCA GTT AGC YAT GAC AGA TAC AAC		
	DENYF-A1-PCR-R2a	CCA ACT ACC GCA ACC ACA CAT GTA CCA TAT TGC RCG ACT YCC		
	DENYF-A1-PCR-R2b	CCA ACT ACC GCA ACC AAC ATG TAC CAT ATG GCT CTG CTR CC		
	DENYF-A1-PCR-R2c	CCA ACT ACC GCA ACC ACC ACA TAT ACC ATA TRG CYC TAC TGC C		
	DENYF-A1-PCR-R2d	CCA ACT ACC GCA ACC ACA TRT ACC ARA TTG CYC GGC TTC C		
	DENYF-A1-PCR-R2e	CCA ACT ACC GCA ACC ACA TRT ACC AKA TRG CAC GGC TTC C		
FLAVI AMP2	DENYF-A2-PCR-F1a	GCC AAC TAC CGC AAC ACG ATG AYA CAG CCG GAT GGG AYA C	NS5	409
	DENYF-A2-PCR-F1b	GCC AAC TAC CGC AAC ACG ATG ACA CMG CMG GMT GGG ACA C		
	DENYF-A2-PCR-F1c	GCC AAC TAC CGC AAC ACG AYG AYA CWG CAG GAT GGG ACA C		
	DENYF-A2-PCR-F1d	GCC AAC TAC CGC AAC ACG AYG AYA CAG CTG GKT GGG ACA C		
	DENYF-A2-PCR-F1e	GCC AAC TAC CGC AAC ACG AYG ACA CRG CAG GYT GGG ACA C		
	DENYF-A2-PCR-F1f	GCC AAC TAC CGC AAC ACG ATG AYA CCG CTG GRT GGG AYA C		
	DENYF-A2-PCR-R1a	CCA ACT ACC GCA ACC AAC RCA RTC ATC TCC RCT GAT TGC C		
	DENYF-A2-PCR-R1b	CCA ACT ACC GCA ACC AAC RCA RTC ATC TCC ACT GAT KGC C		
	DENYF-A2-PCR-R1c	CCA ACT ACC GCA ACC AAC RCA ATC ATC YCC GCT RAT GGC C		
	DENYF-A2-PCR-R1d	CCA ACT ACC GCA ACC AAC RCA ATC RTC TCC ACT RAT TGC C		
	DENYF-A2-PCR-R1e	CCA ACT ACC GCA ACC AAC RCA RTC GTC TCC ATT CAC CGC C		
