# Supplementary material for: A Multiplex PCR/LDR Assay for the Simultaneous Identification of Category A Infectious Pathogens: Agents of Viral Hemorrhagic Fever and Variola Virus
Source: PLoS One. 2015 Sep 18;10(9):e0138484. doi: 10.1371/journal.pone.0138484 (PMC4575071; doi:10.1371/journal.pone.0138484)
Supplement: S2 Table — Primer names indicate the virus detected as well as the amplicon on which the LDR primer pairs were designed. For example, the first primer was designed to detect RESTV and was designed on a nucleotide position on amplicon 1. When more than one primer pair was designed for a particular virus, they were indicated with different numbers 1,2,3 etc. When multiple primers were designed at any LDR position an alphabetical suffix is included (for example, “A” or “B”). An amino blocking group (Blk) was used at the 5’end of the upstream primers and downstream primers were labeled with Cy-3 at the 3’ end. The first 20 nucleotides of the upstream primer (underlined) are the zip-code complements. (PDF) [file pone.0138484.s004.pdf]

Upstream Primer		Downstream Primer	
Primer Name	Sequence	Primer Name	Sequence
ER-A1-LDR-UP1	Blk-<u>TTGGCCTGTTCCCGCTCGCT</u><br>GAAAAAGTCCAGCGACAAATTCAGGTT	ER-A1-LDR-DP1	CATGCAGAACAGGGTTTAATTCAATATCCCAC
ER-A1-LDR-UP2	Blk-<u>CCTGCGCTGCGTGGTGTTCC</u><br>ATCCACCAGGGTATGCATATGGTAGCT	ER-A1-LDR-DP2	GGCCACGATGCCAATGATGCTGTC
ER-A1-LDR-UP3	Blk-<u>TCGCGGACGGTGAGCACTGC</u><br>GTTAATGCATTTAAGGCCGCCCTA	ER-A1-LDR-DP3	AGCTCACTTGCTAAGCATGGGGARTATGC
ER-A2-LDR-UP4	Blk-<u>TTCCCGCTGGTGCCACTCCG</u><br>TCGCTATGAGTTCACTGCACCATTTATT	ER-A2-LDR-DP4	GAGTACTGCAACCATTGCTATGGTGTGCGT
ER-A2-LDR-UP5	Blk-<u>CGCTCGCTTTGGCCTGCTGC</u><br>TTAATCCCGCAGTGTTACATGCATGTA	ER-A2-LDR-DP5	AGTGATTATTATAATCCGCCTCACAATGTTAATCTTAGC
ER-A2-LDR-UP6	Blk-<u>CGCTGCGTGAGGGCTCGGAC</u><br>CGAGTTCGTACCGAGGGCACTTA	ER-A2-LDR-DP6	GGAGGCATAGAGGGATTACAACAAAAACTGTGT
EZ-1-LDR-UP7	Blk-<u>TTCCACCCGCGTCGTCCTGC</u><br>TTGAGAAGGTTCAAAGGCAAATTCAAGTA	EZ-A1-LDR-DP7	CATGCAGAGCAAGGACTGATACAATATCCAACA
EZ-A1-LDR-UP8	Blk-<u>GACCGCTCAGCAGACCGCCA</u><br>CAAGGGATGCACATGGTTGCC	EZ-A1-LDR-DP8	GGGCATGATGCCAACGAYGCTGT

EZ-A1-LDR-UP9	Blk-AGCACGCTTCCGGGACCCAC GAGGTGAACTCCTTTAAGGCTGCACTC	EZ-A1-LDR-DP9	AGCTCCCTGGCCAAGCATGGAGAGTAT
EZ-A2-LDR-UP10	Blk-AGGCGGTGTTGGCTCGAGGC TTGCATTTAGATATGAGTTTACAGCACCTTTTAT A	EZ-A2-LDR-DP10	GAATATTGCAACCGTTGCTATGGTGTTAAGAATGT
EZ-A2-LDR-UP11	Blk-GCGTGTCTCCTGTCCGGCCA CATTATACAATCCCACAGTGTTATATGCATGTC	EZ-A2-LDR-DP11	AGTGATTATTATAATCCACCACATAACCTCACACTGGT
EZ-A2-LDR-UP12	Blk-TTGGGCTCGTGAAGGCAGGC GGGCCTAGTTCATACAGGGGTCATATG	EZ-A2-LDR-DP12	GGAGGGATTGAAGGACTGCAACAAAAACTCT
ES-A1-LDR-UP13	Blk-GAGGTTCCCGCTTCGCCCAC GGAAAAAGTACAAAGGCAGATTCAGGTC	ES-A1-LDR-DP13	CATGCAGAACAAGGGCTCATTCAATATCCA
ES-A1-LDR-UP14	Blk-GGTGCCACGTGATCGCTCCG AGGGGATGCACATGGTCGCA	EZ-A1-LDR-DP14	GGCCATGATGCGAATGACACAGTAATATCTAATT
ES-A1-LDR-UP15	Blk-TTGGAGCAGGTGCGTCGCGT GAGGTCAGTTCATTCAAGGCAGCTCTT	ESA1-LDR-DP15	GGCTCACTTGCCAAGCATGGAGAATATGT
ES-A2-LDR-UP16	Blk- CTGCAGGCGCGTGAGGGAGG CAGGTATGAATTCACAGCTCCCTTCATC	ESA2-LDR-DP16	AAATATTGCAACCAATGCTATGGGGTTCGT
ES-A2-LDR-UP17	Blk-GCCATCCGCGCTCAGTCCAC CTAATTCCGCAATGTTACATGCATGTT	ES-A2-LDR-DP17	AGTGATTATTATAACCCACCACATAATGTAACCTTAGAGAAT AGT
ES-A2-LDR-UP18	Blk-GGTGGGACGCGTCTGCCTCG GGACCAAGTGCTTATAGAGGCCACCTT	ES-A2-LDR-DP18	GGCGGTATTGAGGGGCTTCAACAAAAGT
M-A1-LDR-UP19	Blk-CGCTCCTGCAGTCTCGGCCA CCAAATTAYGTGGGYGATTTAAACYTAGAT	M-A1-LDR-DP19	GAYCAGTTYAAAGGGAATGTCTGCCAYGC
M-A1-LDR-UP20	Blk-CTCGTCCGCAGTCGTCCGCT GCATGGCTGCCTCTTGGGATTAT	M-A1-LDR-DP20	GAGCAATTTTGAATATCCTTTAGCTCATACTGTGGC

M-A1-LDR-UP21	Blk-TCGCTTGGGTCTTCGCAGGC AATCCCRGCACACCCACTCAGAAT	M-A1-LDR-DP21	GTTGCGTGAAGGAAATCAAGCTTTTATTCAGAA
M-A2-LDR-UP22	Blk-CTGCCTCGGCGTCGCTGCTC AATCTTGACCTTAATTCCACAGCACGAT	M-A2-LDR-DP22	GGATAAAAGAACCCAGTGTTGGGGGCTGT
M-A2-LDR-UP23	Blk-TTGGGACCCTCGGGACTCGC AACTTTGTTTTCCAYATACCAAATACTGGAAT	M-A2-LDR-DP23	GACATTGTTGCATCATTTAAAGTCTAACTTCGTTGTT
M-A2-LDR-UP24	Blk-TTGGCTGCCGCTCGCTTTCC ATTGATTCCTGGATTTAGATCYATTGTTCATAT	M-A2-LDR-DP24	GCTWTCAGAATGGYTGCTCCTRGAGGTC
CC-A1-LDR-UP36	Blk-TCGCGACCGTCTGCTCAGGC TCCAGATGGCTAGTGCCACYGATGAT	CC-A1-LDR-DP36	GCACAAAAGGATTCCATCTACGCATCTGC
CC-A1-LDR-UP37	Blk-TCGCGACCGTCTGCTCAGGC CCAGATGGCCAGTGCTACAGACGAT	CC-A1-LDR-DP37	GCACAAAAGGAYTCCATCTATGCWKCTGC
CC-A1-LDR-UP38	Blk-TCGCGACCGTCTGCTCAGGC AGATGGCYRGTGCAACCGATGAT	CC-A1-LDR-DP38A	GCACARAAAGAYTCCATCTATGCATCTGC
		CC-A1-LDR-DP38B	GCACAGAAGGACTCTATATATGCATCTGC
CC-A1-LDR-UP39	Blk-CGCTGGACCGCTAGGCACCC AAATGCAGGAACCATTAAATCWTGGGAT	CC-A1-LDR-DP39A	GARAGTTAYACTGAGCTGAAAGTTGARGTTCCCA
		CC-A1-LDR-DP39B	GAGAGTTATACCGAGTTGAAGGTTGAAGTTCCCA
CC-A1-LDR-UP40	Blk-CGCTGGACCGCTAGGCACCC AAATGCAGGAACTATCAAGTCTTGGGAT	CC-A1-LDR-DP40	GAGAGCTACACTGAACTTAAGGTTGGTGTCCCC
CC-A1-LDR-UP41	Blk-CGCTGGACCGCTAGGCACCC AAAYACAGGAACCATTAAGTCTTGGGAT	CC-A1-LDR-DP41	GAGAGCTAYACTGAGCTGAAAGTGGATGTGC

CC-A1-LDR-UP42	Blk-CGCTGGACCGCTAGGCACCC GGTTTGAAAAGAACACGAGCACTATCAAAT	CC-A1-LDR-DP42	CCTGGGACGAGAGCTACACTGAGCTGAAGT
CC-A1-LDR-UP43	Blk-TTGGGCGTTGGTGCGTCCTG GAGGAAAGACATAGGCTTCCGTGTCAAT	CC-A1-LDR-DP43	GCAAACACRGCAGCTTTRAGYAACAAGGT
CC-A1-LDR-UP44	Blk-TTGGGCGTTGGTGCGTCCTG GAGGAAGGAYATWGGYTTCCGTGTCAAT	CC-A1-LDR-DP44A	GCAAATACGGCAACCCTGAGCCATAAAGT
		CC-A1-LDR-DP44B	GCTAATACGGCAGCCTTGAGCAACAAAGT
CC-A1-LDR-UP45	Blk-TTGGGCGTTGGTGCGTCCTG GAGGAAGGACATAGGCTTCCGCGTTAAT	CC-A1-LDR-DP45	GCAAACACAGCGGCCCTAAGCAACAAAGT
CC-A1-LDR-UP46	Blk-TTGGGCGTTGGTGCGTCCTG GAGAAAGGACATAGGTTTCCGCATCAAT	CC-A1-LDR-DP46	GCTAACACAACAGCGCTGAGCAACAAAGT
CC-A2-LDR-UP47	Blk-GCTCCAGTTCCGGGTGCGCT TGAAGAAGGCACTYYTGAGCACHCCAAT	CC-A2-LDR-DP47A	GAAGTGGGGGAAGAAGCTTTATGAGCTYTTY
		CC-A2-LDR-DP47B	GAARTGGGGAAAGAAGCTTTATGAGCTYTTYGT
CC-A2-LDR-UP48	Blk-GCTCCAGTTCCGGGTGCGCT TGAAGAAAGCTCTGCTCAGCACTCCCAT	CC-A2-LDR-DP48	GAAGTGGGGRAAGAARCTTTATGARCTCTTTGT
CC-A2-LDR-UP49	Blk-GCTCGAGGCAGTCCACGCGT GTGCTRACAGCYGGCAGAATCAGTGARAT	CC-A2-LDR-DP49	GGGWGTCTGCTTTGGRACAATCCCTGTGT
CC-A2-LDR-UP50A	Blk-GCTCGAGGCAGTCCACGCGT GTGTTAACAGCCGGCAGAATCAGTGAGAT	CC-A2-LDR-DP50	GGGTGTYTGCTTYGGAACCATCCCT
CC-A2-LDR-UP50B	Blk-GCTCGAGGCAGTCCACGCGT GTGCTGACAGCTGGCAGAATTAGTGAGAT		
CC-A2-LDR-UP51	Blk-GCTCGAGGCAGTCCACGCGT GTGCTGACAGCTGGTAGGATCAGCGAAAT	CC-A2-LDR-DP51	GGGTGTCTGCTTTGGGACAATTCCT

CC-A2-LDR-UP52	Blk-GCGTGAGGTTGGGAGGGCGT CAGGRTTCAACATACAGGACATGGAYATT	CC-A2-LDR-DP52	GTDGCCTCTGAGCACCTRCTGCACCA
CC-A2-LDR-UP53A	Blk-GCGTGAGGTTGGGAGGGCGT CAGAAAAAAGGATTTGTAATAAAAGATATGGA CATT	CC-A2-LDR-DP53	GTGGCCTCTGARCATCTGCTRCAYCA
CC-A2-LDR-UP53B	Blk-GCGTGAGGTTGGGAGGGCGT GGGCTTTGACATAAAGGACATGGACATC		
RVF-A1-LDR-UP54	Blk-TTGGTTGGGAGGCTGCGGTG ATGGCCARCTCAGCACTGCACAT	RVF-A1-LDR-DP54	GAGGTTGTGCCCTTTGCAGTGTTTAAGA
RVF-A1-LDR-UP55	Blk-CGTCGACCTTCCGCCAACCC GGAMGCAGCATTTTGCTCTGCTTAT	RVF-A1-LDR-DP55A	GAATGCACTGCTCAGTATGCCAATGCC
		RVF-A1-LDR-DP55B	GAGTGYACTGCTCARTAYGCCAATGCC
RVF-A1-LDR-UP56	Blk-CGTCGCTCTGGTTCGCTCGC GGTCTGGAAKAARCCTTTATGTGTAGGGTAT	RVF-A1-LDR-DP56	GAGAGAGTRGTTGTGAAGAGAGAACTCKCTGCY
RVF-A2-LDR-UP57A	Blk-GGTGACCCGTGACGCTGCCA CRAGTCGGACTKGGAGACTTTGCAT	RVF-A2-LDR-DP57	CAAAYGTKGCACCTCCACCAGCRAAGT
RVF-A2-LDR-UP57B	Blk-GGTGACCCGTGACGCTGCCA GAGTCSGGCTKGGAGACTTTGCAT		
RVF-A2-LDR-UP58A	Blk-AGGCTTGGCGCTCGTCACCC GTTCCCCAATCTAAAAGAAGCCATAT	RVF-A2-LDR-DP58A	CCTGGCCTCTTRGAGARCCCTCMCT
RVF-A2-LDR-UP58B	Blk-AGGCTTGGCGCTCGTCACCC GTTCCCCAACTCAAAAGAAGCCATAT	RVF-A2-LDR-DP58B	CCTGGCCTCTTGGAGAACCYTMMTT

		RVF-A2-LDR-DP58C	CCTGGCCTCTTGGWGAACCCTCWCY
RVF-A2-LDR-UP59	Blk-GTCTCCACCGCTGCCATCGC GCAATGAGGAGYTGCAAGATYRCCAAT	RVF-A2-LDR-DP59A	GATCTWGAGGACTCCTTTGYTGGCTTACACAGT
		RVF-A2-LDR-DP59B	GATYTAGAAGACTCCTTTGTTGGCYTRCACAGT
VAC-A1-LDR-UP60	Blk-CGTCGTGAGCCATCGCCTGC CTAAAAGAATTRGGTTCTTTCGATTTGGAA	VAC-A1-LDR-DP60	AACGTCAACGTGTATCCTGGAGTATGGAATACATAC
VAC-A1-LDR-UP61	Blk-GCCATCGCCCACAGCAGGAC GCCATGTTAAACACCTATGGAGAAGGG	VAC-A1-LDR-DP61	TTCGATTATCTGTATCAGCCTTGTGATCTTCTCGT
VAC-A2-LDR-UP62	Blk-AGCAAGGCGACCGGACGACC ATTGAACGGTATCTTCTTAATCTCTGGTTCTAG	VAC-A2-LDR-DP62	TTCCGCATTAAATGATGAAACTAAGTCACTATTTTTA
VAC-A2-LDR-UP63	Blk-GGACGAGGAGGCCGCTTGGT TACATCACCTCTAACATCATCATTTACCAGA	VAC-A2-LDR-DP63	ATACTGATCTTCTTTTGTCGTAAATACATGTCTAATGTGTT
VAC-A2-LDR-UP64	Blk-CCTGGCCAGACCTTCCGCGT GAAGGATAAACTCGTACTAATCTCTTCTTTAAC AGC	VAC-A2-LDR-DP64	CTGTTCAAATTTATATCCTATATACGAAAAAATAGCAACCAG T
VAR-A1-LDR-UP65	Blk-CCTGCTCGTCGCTCCGTTGG AAGATTCCTATCTCTGATTATACAGGTTATGAC GAT	VAR-A1-LDR-DP65	TTCAATCAAACAAAAAAGCTCAATAAGATGACTGTAGAA
VAR-A1-LDR-UP66	Blk-GTGATCGCCGTCCCTGTCCG TATCGAGGCCGCTCGTGGATACTTA	VAR-A1-LDR-DP66	TGCGAAGCCATGTTAAACACCTATGGAGAAGT

VAR-A2-LDR-UP67	Blk-TTCCGAGGCCTGACCCGACC ATAGATTTTTCATATTGAAYGGTATCTTCTTGATT	VAR-A2-LDR-DP67	TCTGGTTCTAATTCCGCATTAAATGATGAAACTAAGT
VAR-A2-LDR-UP68	Blk-GGTGTGGTCTCGCGCTTCCG ATACGTCATTTCATCTGTAGTATTCTTGTCATTRAG	VAR-A2-LDR-DP68	TGATAAACTTGTGCTAATCTCTTCTTTAACAGTCTGTTCA
VAR-A2-LDR-UP69	Blk-TCCGTTCCGCCAGAGGGTGA CGAAAAAATAGCAACCAATGTTTGATCATT	VAR-A2-LDR-DP69	CGCGTCAATATTCTGTTCTATTGTARTGTATAACAATCTTATATCTT
L-A1-LDR-UP70	Blk-TCGCCGTCCGCTGTCTTTGG ATTTGCCTCATRAAATTGTCAAYAGCAT	L-A1-LDR-DP70	CACTAGTRCTCACWACTCTTTCTTCMACCATGGT
L-A1-LDR-UP71	Blk-TCGCCGTCCGCTGTCTTTGG ATTTGCCKCATGAARTTGTCAACWGCAT	L-A1-LDR-DP71	CACTAGTRCTCACCACCCTTTCTTCCACCATAGT
L-A1-LDR-UP72	Blk-GACCAGGCCTCGACCCACCC CACAGTTAACATCCAACGCTACACAGAGAT	L-A1-LDR-DP72	CYAGAAAYTTTATTCTRGGTGACCACTTCATTTTGT
L-A1-LDR-UP73A	Blk-GACCAGGCCTCGACCCACCC CACAACTGACATCTAGTGCCACACAAAGAT	L-A1-LDR-DP73	CYAGRAATTTTATCCTGGGTGACCACTTCAT
L-A1-LDR-UP73B	Blk-GACCAGGCCTCGACCCACCC CACAATTCACATCCAGTGCCACACAGAGT		
L-A1-LDR-UP74	Blk-GCCACGCTGCCAGGACTCCG CCAACCCTATCCTCCAATAGYTTTGAT	L-A1-LDR-DP74	GCAGTTGGCTTYAAGGGAAAATARTCACAAGGT
L-A1-LDR-UP75	Blk-GCCACGCTGCCAGGACTCCG CCRATCCTGTCCTCCAGCAATTTAGAT	L-A1-LDR-DP75	GCWGTTGGCTTTAGAGGGAARTAATCACAAGGT
L-A1-LDR-UP76	Blk-GCCACGCTGCCAGGACTCCG CCCACTCTATCTTCAAGTAATTTTGAT	L-A1-LDR-DP76	GCGGTCGGCTTCAAAGGGAAATAATCACAAGGT

L-A2-LDR-UP77	Blk-TCCGGTCTTGGTCGCTTCGC<br>GCAATRGARCTCTGAAATGCAGATTT	L-A1-LDR-DP77A	GTTGATAGCTTCAGACAGCAATTTTTGCGC
		L-A1-LDR-DP77B	GTTGATRGCYTCAGATARTAATTTCTGTGC
		L-A1-LDR-DP77C	GTTTATGGCTTCTGATAGCAGTTTCTGTGC
L-A2-LDR-UP78	Blk-GTCTTCGCGGTGGGTGCCTG<br>TTCTCRTCATCTAGGTTCACAGCACCT	L-A1-LDR-DP78A	GAATATATTATTTTTTGCCTTAAAACCATTCTCAGAGGATGT
		L-A1-LDR-DP78B	GAATATATTATCTTTTGYCTCAACACCATYCTTAARGGATGT
L-A2-LDR-UP79	Blk-GTCTTCGCGGTGGGTGCCTG<br>TTCATCATCAAGGTTTACGGCACCT	L-A2-LDR-DP79	GAGTATATAACCTTCTGTCTCAAAACCATTCTTAGAGGGTGT
L-A2-LDR-UP80A	Blk-GCGTTTGGTTGGCTGCGGAC<br>CGGTTRCCRTCTACCCAGTCTCTAACAT	L-A2-LDR-DP80	CTGTCTCGCARTTCAAWARGAATGGGTCAAT
L-A2-LDR-UP80B	Blk-GCGTTTGGTTGGCTGCGGAC<br>TTGCCATCTACCCAATCTCTGACAT		
L-A2-LDR-UP81	Blk-GCGTTTGGTTGGCTGCGGAC<br>CGGTTGCCGTCCACCCAGTCTTTTACAT	L-A2-LDR-DP81	CTGTTTCACAGTTCAGAAGGAATGGGTCAAT
DEN-A1-LDR-UP82A	Blk-GCTCACCCGGACGCCACTGC<br>GTGTTYAAAGAGAAAGTTGACACRCGCACRC	DEN-A1-LDR-DP82A	CAARAGCRAAACGRGGCACAGCACA
DEN-A1-LDR-UP82B	Blk-GCTCACCCGGACGCCACTGC<br>CGCGTTTTCAAAGARAAAGTRGACACGAGAACY<br>C	DEN-A1-LDR-DP82B	AAGAACCGAARGAAGGCACRAAGAAACTRAT
DEN-A1-LDR-UP82C	Blk-GCTCACCCGGACGCCACTGC<br>TYTTYAAAGAGAAAGTGGACACCAGGACRC	DEN-A1-LDR-DP82C	CCAGGTCCATGCCAGGAACAAGAAGGGT
DEN-A1-LDR-UP82D	Blk-GCTCACCCGGACGCCACTGC<br>GTGTTCAARGAGAAGGTGGAYACCAGAACWC	DEN-A1-LDR-DP82D	CTARRCCCATGCCAGGRACAAGAAAGGT

		DEN-A1-LDR-DP82E	CACARCCAAAACCCGGYACACGART
		DEN-A1-LDR-DP82F	CACAGCCMAAACYAGGCACACGAGT
DEN-A1-LDR-UP83A	Blk-TCCGCTGCCCACTTCCCTCG GCCATYGGAGCAGTGGTTCGTTGAYG	DEN-A1-LDR-DP83A	AAAAYCAATGGAACTCAGCAAAAGARGCAGT
DEN-A1-LDR-UP83B	Blk-TCCGCTGCCCACTTCCCTCG GCWATWGGAGCAGTGGTTCGTTGATG	DEN-A1-LDR-DP83B	AGAACAARTGGAARTCGGCWCGTGAGGC
DEN-A1-LDR-UP83C	Blk-TCCGCTGCCCACTTCCCTCG GCAAYGCAGCYTTGGGNGCCATATTCACTGATG	DEN-A1-LDR-DP83C	AGAACCAATGGGAYAGYGCGARAGCTGCT
DEN-A1-LDR-UP83D	Blk-TCCGCTGCCCACTTCCCTCG GCYATGGGAGCYGTTTTCACAGAGG	DEN-A1-LDR-DP83D	AGAACCAATGGGACAGTGCYARAGCTGCYGT
DEN-A1-LDR-UP83E	Blk-TCCGCTGCCCACTTCCCTCG GCTATGGGCGCYGTHTTYACAGAG	DEN-A1-LDR-DP83E	AACAGGGATGGRCATCAGCCAGTGAAGC
DEN-A1-LDR-UP83F	Blk-TCCGCTGCCCACTTCCCTCG CAGCCATAGGCGCRGTCTTYCAGGARG		
DEN-A1-LDR-UP84A	Blk-GTCTGCCACTGCGCTCGCCA GAGAGGGAGCTTCAYAAACAGGGAAARTGYG	DEN-A1-LDR-DP84A	CYACRTGTGTYTACAACATGATGGGGAAGAGA
DEN-A1-LDR-UP84B	Blk-GTCTGCCACTGCGCTCGCCA CARGGAAAGRAATCTYCATCTTGAAGGAAAGTG TG	DEN-A1-LDR-DP84B	ARACATGTGTGTACAACATGATGGGAAARAGAGARAA
DEN-A1-LDR-UP84C	Blk-GTCTGCCACTGCGCTCGCCA CGTGAACTCCATAAAYTGGGCAARTGYG	DEN-A1-LDR-DP84C	GAAGCTGYGTYTAYAACATGATGGGCAAGAGA
DEN-A1-LDR-UP84D	Blk-GTCTGCCACTGCGCTCGCCA AAGGAAAGRAAYCTNCATCTTGAAGGGAAATG TG	DEN-A1-LDR-DP84D	AATCRTGYGTCTAYAACATGATGGGAAAACGT

DEN-A2-LDR-UP85A	Blk-CGCTTTCCCTGCAGGCCTCG<br>AACATGCYCTAYTGGCTATGGCAATYTTTA	DEN-A1-LDR-DP85A	ARCTAACATACCAAAAYAARGTGGTAAGGGT
DEN-A2-LDR-UP85B	Blk-CGCTTTCCCTGCAGGCCTCG<br>AACATGCYCTAYTGGCCACGGCAATCTTA	DEN-A1-LDR-DP85B	AGYTRACCTACCAAAATAARGTGGTAAGGGT
DEN-A2-LDR-UP85C	Blk-CGCTTTCCCTGCAGGCCTCG<br>ACACAAGAAAYTWGCYGAGGCCATTTTCA	DEN-A1-LDR-DP85C	AGYTAACMTACCAAAAYAAGGTGGTAAGGGT
DEN-A2-LDR-UP85D	Blk-CGCTTTCCCTGCAGGCCTCG<br>ACACAAGAAACTAGCCGAGGCCATWTTYA	DEN-A1-LDR-DP85D	RAYTAACGTAYCAAAACAAGGTGGTGCGTGT
DEN-A2-LDR-UP85E	Blk-CGCTTTCCCTGCAGGCCTCG<br>ACACAAGAAATTGGCCGAGGCTATTTTCA	DEN-A1-LDR-DP85E	AGCTCACRTACCARAAYAAAGTGGTCAAAGTTCAA
DEN-A2-LDR-UP85F	Blk-CGCTTTCCCTGCAGGCCTCG<br>CCTGAACACAGGCAGYTAGCRAACGCTATATTYA	DEN-A1-LDR-DP85F	AACTGACTTACCAAAATAAAGTGGT
DEN-A2-LDR-UP85G	Blk-CGCTTTCCCTGCAGGCCTCG<br>CCTGAACACAGACARTTAGCGAACGCYATATTYA	DEN-A1-LDR-DP85G	AAYTAACYTATCAAAACAAAGTGGT
DEN-A2-LDR-UP85H	Blk-CGCTTTCCCTGCAGGCCTCG<br>CCYCACCACAAGAYCCTAGCCAAAGCCATYTTCA		
DEN-A2-LDR-UP85I	Blk-CGCTTTCCCTGCAGGCCTCG<br>CCTCATCATAARATCYTAGCYAAAGCCATTTTCA		
DEN-A2-LDR-UP86A	Blk-GCTCTCGCACCCGGACCTCG<br>GTGCARAGACCAGCAAAAAAYGGAACYGT	DEN-A1-LDR-DP86A	GATGGATGTYATATCCAGRCGTGACCARAGA

DEN-A2-LDR-UP86B	Blk-GCTCTCGCACCCGGACCTCG<br>CGTGTRCAAAGACCAACACCAAGAGGMACAGT	DEN-A1-LDR-DP86B	AATGGAYATYATATCGAGAAGAGACCAAAGAGGYA
DEN-A2-LDR-UP86C	Blk-GCTCTCGCACCCGGACCTCG<br>TCAACGACCRACTCCAAMRGGCACGGT	DEN-A1-LDR-DP86C	RATGGACATCATATCGAGAARAGACCAAAGAGGCA
DEN-A2-LDR-UP86D	Blk-GCTCTCGCACCCGGACCTCG<br>TYCTCAGACCCACACCGARAGGAGCRGT	DEN-A1-LDR-DP86D	RATGGACATYATATCTAGRAAAGACCAAAGAGGCA
		DEN-A1-LDR-DP86E	AATGGAYATTATATCTAGGAAAGACCAAAGGGGCA
		DEN-A1-LDR-DP86F	VATGGAYATYATATCCAGGAAAGACCAAAGAGGTA
DEN-A2-LDR-UP87A	Blk-CTGCCCTGCGTCGCTCCCAC<br>GARGCCCAACTRATAAGACARATGGAGTCTGAG<br>G	DEN-A1-LDR-DP87A	GAATYTTTTYACCCAGCGAATTRGAGACCC
DEN-A2-LDR-UP87B	Blk-CTGCCCTGCGTCGCTCCCAC<br>GAGGYCCAACTAATAAGACAAATGGAGTCTGA<br>RG	DEN-A1-LDR-DP87B	GAGTCTTYAAAARCATCCAGCACCTGACAGYC
DEN-A2-LDR-UP87C	Blk-CTGCCCTGCGTCGCTCCCAC<br>GCCCAAYTAATYAGACAGATGGAAGGAGAAG	DEN-A1-LDR-DP87C	GAATYTTYAAAAGCATTCAGCRCCTGACAGTC
DEN-A2-LDR-UP87D	Blk-CTGCCCTGCGTCGCTCCCAC<br>CAACTRATTAGGCAGATGGAGGGGGAAG	DEN-A1-LDR-DP87D	GYGTGTTGTCRAAGRCAGACCTCGAGAACCCT
DEN-A2-LDR-UP87E	Blk-CTGCCCTGCGTCGCTCCCAC<br>GCCCAGTTRRTCAGACARATGGAAGGAGAAG	DEN-A1-LDR-DP87E	GTGTGCTGWCAAAGGCAGACCTCGAGAA
DEN-A2-LDR-UP87F	Blk-CTGCCCTGCGTCGCTCCCAC<br>CTYATCCGCCARATGGAAGCTGARG	DEN-A1-LDR-DP87F	GAGTYATCACACRAGAYGACATGCAGAACCCAA
YF-A1-LDR-UP88A	Blk-GTGAAGGCCGTCGGACCGCT<br>CAAGGATCCACCMGCRGGAACYAGAAAGTTG	YF-A1-LDR-DP88A	TCAAYARGTGGCTSTTCCGCCACCT

YF-A1-LDR-UP88B	Blk-GTGAAGGCCGTCGGACCGCT TCCTGCTGGAACHAGGAARATCATGARGGTGG	YF-A1-LDR-DP88B	TAAACCGWTGGTTGTTTCGACAYCTRGC
		YF-A1-LDR-DP88C	TGAATAGATGGTTGTTTCGGCATCTGTC
YF-A1-LDR-UP89A	Blk-CGCTCTCGCTGCCTCGGGAC GTGYACAAAGGAAGAATTYATTGCAAARGTCC	YF-A1-LDR-DP89A	GCAGTCATGCTGCGRTAGGRGGCCT
YF-A1-LDR-UP89B	Blk-CGCTCTCGCTGCCTCGGGAC GCACAAAGGARGARTTCATAGCCAAGGTGC	YF-A1-LDR-DP89B	GGAGCCATGCCGCAGTGGGGGC
YF-A1-LDR-UP89C	Blk-CGCTCTCGCTGCCTCGGGAC GCACCAAAGAAGAGTTCATTGCCAAAGTGC	YF-A1-LDR-DP89C	GMAGYCAYGCAGCCATTGGAGCTTACCT
YF-A1-LDR-UP90A	Blk-AGGCCGCTGACCGTGACTGC AAGACAGCCAATGAAGCRGTYCAAGACCC	YF-A1-LDR-DP90A	YAAGTTTTGGGAGATGGTTGAYGCTGAGC
YF-A1-LDR-UP90B	Blk-AGGCCGCTGACCGTGACTGC CGGCCAATGAGGCAGTCCAGGATCC	YF-A1-LDR-DP90B	AAAGTTTTGGGAGATGGTCGATGCAGAAC
YF-A1-LDR-UP90C	Blk-AGGCCGCTGACCGTGACTGC TGCCAAYGAGGCYGTCCARGACCC	YF-A1-LDR-DP90C	RAAGTTYTGGGAAYTGGTGGATGAAGAAAG
YF-A2-LDR-UP91A	Blk-ACCCTCCGTCCGTTGGCGTC GACCTRGAYGATGARCAGGAGATCATGAGCT	YF-A2-LDR-DP91A	ACATGAACGCTGAGCAGAGGAAACTGGC
YF-A2-LDR-UP91B	Blk-ACCCTCCGTCCGTTGGCGTC GACCTGGATGACGAGCAAGAGATCATGAGTT	YF-A2-LDR-DP91B	ACATGAGCCCTGAACAAAGGAAGCTGGC
YF-A2-LDR-UP91C	Blk-ACCCTCCGTCCGTTGGCGTC GCAGACCTTGATGATGAACAGGARATCYTGAAY T	YF-A2-LDR-DP91C	ACATGAGCYCACATCACAAAAAACTGGCACA
YF-A2-LDR-UP92A	Blk-GGTGTCCGGGTGTCGCGTGA CCTYCGACCAGCACCAGGWGGCAA	YF-A2-LDR-DP92A	GGCYTTCATGGACATCATCAGYMGGAGAGA

YF-A2-LDR-UP92B	Blk-<u>GGTGTCCGGGTGTCGCGTGA</u> TGAGGCCAGCCCCAGGAGGCAA	YF-A2-LDR-DP92B	AGCCTACATGGATGTCATAAGYCGRCGAGAYCA
YF-A2-LDR-UP92C	Blk-<u>GGTGTCCGGGTGTCGCGTGA</u> GTGCTGAGACCARCMCCAGGMGGGAA		
YF-A2-LDR-UP93A	Blk-<u>CCTGGCTCGCTCTCCGCTCG</u> CACYATCACCAATYTGAAAGTYCAGCTCATAAGGA	YF-A2-LDR-DP93A	TGGCTGAAGCTGAGATGGTAATCAACCATC
YF-A2-LDR-UP93B	Blk-<u>CCTGGCTCGCTCTCCGCTCG</u> CACCATAACCAATCTAAAAGTCCAGCTCATAAGAA	YF-A2-LDR-DP93B	TGGCTGAAGCTGAAATGGTGATTAACCATC
YF-A2-LDR-UP93C	Blk-<u>CCTGGCTCGCTCTCCGCTCG</u> CACYATCACCAACYTGAAAGTCCAATTGATCA	YF-A2-LDR-DP93C	GRATGGCAGAAGCAGAGAWKGTGATACATCA

.
